# MicroRNA Profiling of Neurons Generated Using Induced Pluripotent Stem Cells Derived from Patients with Schizophrenia and Schizoaffective Disorder, and 22q11.2 Del

**DOI:** 10.1371/journal.pone.0132387

**Published:** 2015-07-14

**Authors:** Dejian Zhao, Mingyan Lin, Jian Chen, Erika Pedrosa, Anastasia Hrabovsky, H. Matthew Fourcade, Deyou Zheng, Herbert M. Lachman

**Affiliations:** 1 Department of Neurology, Albert Einstein College of Medicine, 1300 Morris Park Ave., Bronx, New York, United States of America; 2 Department of Genetics, Albert Einstein College of Medicine, 1300 Morris Park Ave., Bronx, New York, United States of America; 3 Department of Psychiatry and Behavioral Sciences, Albert Einstein College of Medicine, 1300 Morris Park Ave., Bronx, New York, United States of America; 4 Department of Neuroscience, Albert Einstein College of Medicine, 1300 Morris Park Ave., Bronx, New York, United States of America; 5 Department of Medicine, Albert Einstein College of Medicine, 1300 Morris Park Ave., Bronx, New York, United States of America; The University of Tennessee Health Science Center, UNITED STATES

## Abstract

We are using induced pluripotent stem cell (iPSC) technology to study neuropsychiatric disorders associated with 22q11.2 microdeletions (del), the most common known schizophrenia (SZ)-associated genetic factor. Several genes in the region have been implicated; a promising candidate is *DGCR8*, which codes for a protein involved in microRNA (miRNA) biogenesis. We carried out miRNA expression profiling (miRNA-seq) on neurons generated from iPSCs derived from controls and SZ patients with 22q11.2 del. Using thresholds of *p*<0.01 for nominal significance and 1.5-fold differences in expression, 45 differentially expressed miRNAs were detected (13 lower in SZ and 32 higher). Of these, 6 were significantly down-regulated in patients after correcting for genome wide significance (FDR<0.05), including 4 miRNAs that map to the 22q11.2 del region. In addition, a nominally significant increase in the expression of several miRNAs was found in the 22q11.2 neurons that were previously found to be differentially expressed in autopsy samples and peripheral blood in SZ and autism spectrum disorders (e.g., miR-34, miR-4449, miR-146b-3p, and miR-23a-5p). Pathway and function analysis of predicted mRNA targets of the differentially expressed miRNAs showed enrichment for genes involved in neurological disease and psychological disorders for both up and down regulated miRNAs. Our findings suggest that: i. neurons with 22q11.2 del recapitulate the miRNA expression patterns expected of 22q11.2 haploinsufficiency, ii. differentially expressed miRNAs previously identified using autopsy samples and peripheral cells, both of which have significant methodological problems, are indeed disrupted in neuropsychiatric disorders and likely have an underlying genetic basis.

## Introduction

Genome wide association studies (GWAS), copy number variation (CNV) analysis and exome sequencing show that schizophrenia (SZ) and other neuropsychiatric disorders, including bipolar disorder (BD) and autism spectrum disorders (ASD), are genetically heterogeneous complex traits. This presents a potential problem in translating molecular and genetic findings into novel therapies that would benefit a large number of patients. Consequently, investigators are applying molecular and genetic data, and bioinformatics to identify common networks onto which many seemingly disparate candidate genes converge. Identifying downstream targets of SZ and ASD candidate genes that function as transcription factors, splicing factors or chromatin remodeling complexes is one potentially useful approach. Another promising area of investigation in this regard is to characterize the role of microRNAs (miRNAs) in disease pathogenesis, considering their common mechanism of biogenesis and converging effect on genes involved in neurogenesis and synaptogenesis [1–9].

MicroRNAs regulate gene expression by inducing double-stranded RNA-mediated decay and translational arrest through base-pair specific interactions with targeted mRNAs, primarily at 3’untranslated regions [10–14]. MicroRNAs are expressed as primary molecules (pri-miRNAs) that are ~70 nucleotides in length that must be processed to form functional, mature miRNAs. The first step in their biogenesis is cleavage by a miRNA processing complex consisting of the proteins DGCR8 and DROSHA, which convert pri-miRNAs into precursor RNAs (pre-miRNAs). These are transported to the cytoplasm where cleavage by DICER occurs, ultimately yielding a single stranded ~22 base mature miRNA that’s incorporated into the RNA-induced silencing complex (RISC), which targets specific mRNAs through a complimentary seed region. Most mRNAs are regulated by more than one miRNA, and any single miRNA can potentially interact with multiple mRNAs [14,15].

Several lines of evidence support a role for miRNAs (actually, their targets) in a subgroup of SZ patients. Replicated GWAS studies, for example, show a strong association to *MIR-137* [16–19]. This miRNA targets other candidate genes identified by GWAS [20,21]. Molecular studies also support a role for miRNAs in SZ. Recently, 28 miRNAs were found to be differentially expressed in the dorsolateral prefrontal cortex (DLPFC) in patients with SZ compared with controls; the mRNA targets of these miRNAs showed enrichment for genes involved in axon guidance and long-term potentiation, processes associated with neuropsychiatric disorders [22,23]. Similarly, an independent study found ~50 miRNAs that were differentially expressed in the DLPFC and superior temporal gyrus in SZ, which targeted and reciprocally down-regulated the expression of mRNAs coding for proteins involved in neurodevelopmental pathways and cell-cell signaling [24].

MicroRNAs have also been considered in the pathogenesis of SZ and other neuropsychiatric disorders that occur in a substantial proportion of patients with velocardiofacial syndrome (VCFS; DiGeorge Syndrome), which is caused by a 22q11.2 del that typically spans ~3 Mb; the *DGCR8* gene maps to the deleted region [25–31]. In addition to SZ, many patients meet criteria for schizoaffective disorder (SAD), ASD, obsessive compulsive disorder (OCD), Tourette Syndrome, depression, anxiety disorder, and rapid cycling BD [32–40]. Conversely, 22q11.2 del is found in ~1% of patients with SZ, usually in the absence of the severe core clinical features characteristic of VCFS, such as cleft palate and congenital heart disease [41]. It is also found in ~4% of patients diagnosed with childhood onset SZ (COS) [42]. The T-box transcription factor *TBX1* is primarily responsible for the major physical manifestations seen in 22q11.2 del [43]. However, the genes underlying the susceptibility to develop neuropsychiatric problems have not been unequivocally identified, although *DGCR8* is a strong candidate [34,44–47]. Mouse *Dgcr8* knockouts show down-regulation of ~25 mature miRNAs in the hippocampus and prefrontal cortex, and heterozygotes have deficits in prepulse inhibition and a spatial working memory–dependent learning task [31]. In addition, *Dgcr8* knockout mice have deficits in the development of excitatory synapses and a reduction of parvalbumin interneurons in the prefrontal cortex [2,48].

In addition to viewing the role of miRNAs in 22q11.2 del from the perspective of the downstream effects of *DGCR8*, haploinsufficiency for individual miRNA genes that map to the deleted region have also been considered in disease pathogenesis. The most well-studied in this regard is *MIR-185*, which has been found to target other SZ candidate genes and influence dendritic spine density in the hippocampus [49,50]. MicroRNA-185 and its targets are also enriched in synapses [7,49,51]. MicroRNA-185 also affects immune developmental pathways, which might contribute to the immunological deficits found in a subset of patients with 22q11.2 del [52,53]. Considering the replicated genetic findings that point to the *HLA* locus in SZ, and the large body of epidemiological and animal studies suggesting that infectious diseases and/or autoimmune phenomena play roles in disease pathogenesis in subgroups of patients with SZ and ASD, an effect of miRNAs on immune function could potentially be of interest in neuropsychiatric disorders [54,55].

Although the 22q11.2 del mouse models have been extremely valuable, it is important to understand the molecular and genetic underpinnings in human neurons for translational research purposes. This is now possible with induced pluripotent stem cell (iPSC) technology, which we and others have been using to model neuropsychiatric disorders *in vitro* [56–68]. Our focus has been on 22q11.2 del syndrome because it is the most common known genetic risk factor in SZ, and one of the most penetrant as well. In order to determine if human neurons derived from patient-specific iPSCs are suitable for modeling the role of miRNAs in 22q11.2 del-associated disorders, and to identify differentially expressed miRNAs, we performed whole transcriptome miRNA sequencing and characterized the potential targets of dysregulated miRNAs.

## Materials and Methods

### Subjects

Controls and patients with 22q11.2 del diagnosed with a psychotic disorder (schizophrenia [SZ], childhood onset schizophrenia [COS], SAD) were recruited from two settings; the Albert Einstein College of Medicine (AECOM) and the National Institutes of Mental Health (NIMH), Child Psychiatry Branch. The study and the consent forms were approved by the AECOM Institution Review Board (IRB) and the NIMH IRB. Consents at AECOM were signed by the subjects at a time when psychotic symptoms were well-controlled with medications. For the NIMH subjects, all participants provided written assent/consent with written informed consent from a parent or legal guardian for minors. Subjects were not disadvantaged in any way if they refused to participate in the study. Consent was obtained by skilled members of the research teams who had received prior human subjects training. The procedure for obtaining informed consent was approved by the AECOM and NIMH IRBs.

The AECOM subjects were diagnosed with VCFS based on typical physical manifestations; the diagnosis was confirmed by FISH. Psychiatric diagnoses were established many years prior to recruitment by the patient’s psychiatrists using non-structured clinical interviews. Upon recruitment, a history of psychosis was confirmed by non-structured clinical interview with the patients and a parent. A more detailed clinical description for the AECOM cohort is provided in S1 Text.

Patients with childhood onset schizophrenia (COS), recruited as part of an NIMH initiative under the leadership of Dr. Judy Rapoport, met DSM-IIIR/DSM-IV criteria for SZ with documented onset of psychosis before age 13 [42]. They were subsequently interviewed for lifetime and current psychiatric disorders using structured psychiatric interviews, with diagnosis confirmed by inpatient, medication-free observation [42].

Controls in both cohorts were assessed by non-structured clinical interviews. There was no personal history of an Axis I diagnosis, and they have never been treated for a psychiatric disorder. It should be noted that we opted not to ascertain controls with 22q11.2 del who have not had psychotic episodes. This was decision was made because of the concern that a subject with 22q11.2 del who never experienced a psychotic episode could be recruited into the study as a young adult as a “control” could potentially onset later in life. Indeed, one of the subjects in this study, SZ_22q11-30, had her first psychotic episode at age 37. Consequently, controls were drawn from the general population.

A summary of the patients and controls used in this study is shown in Table 1.

**Table 1:** Demographics of subjects used for generating iPSCs.

ID	age/sex	diagnosis
**ctrl_iPSC1**	**29/F**	**control**
**ctrl_iPSC2**	**58/M**	**control**
**ctrl_iPSC5**	**32/M**	**control**
**ctrl_iPSC6**	**46/M**	**control**
**ctrl_553**	**31/M**	**control**
**ctrl_690**	**27/M**	**control**
**SZ_iPSC15**	**31/M**	**SAD/VCFS**
**SZ_22q11-30**	**41/F**	**SZ/VCFS**
**SZ_1804**	**25/F**	**COS**
**SZ_1220**	**31/F**	**COS**
**SZ_22q11-10**	**37/M**	**SAD/VCFS**
**SZ_22q11-60**	**25/M**	**SAD/VCFS**

Age refers to the age at time study was carried out. Abbreviations are Schizophrenia (SZ), Schizoaffective Disorder (SAD), Childhood Onset Schizophrenia (COS), velocardiofacial syndrome (VCFS). See S1 Text for clinical details.

### Development of iPSCs from skin fibroblasts; generating iPSC lines

iPSC lines were generated from fibroblasts obtained from skin biopsies performed by board-certified physicians. The procedure for growing fibroblasts in preparation for reprogramming into iPSCs is detailed in S1 Text. Briefly, iPSC reprogramming was carried out by nucleofection. One vial of cells was thawed out and placed in a T75 flask in DMEM/F12 supplemented with 10% FBS and fed every 2 days. Cells were grown to ~50% confluence (~4–5 days), after which they were trypsinized and subjected to nucleofection (~6 x10^5^ cells). Reprogramming was carried out using an Amaxa 4D-Nucleofector (P2 Primary Cell Kit from Lonza catalog# V4XP-2012, Program FF-135) with non-integrating plasmids containing *OCT4*, *SOX2*, *KLF4*, *L-MYC*, *LIN28*, and a p53 shRNA vector (Addgene Cat. # 27077, 27078, 27080), according to Okita et al., with some modifications [63,64,69]. iPSCs were maintained on Matrigel plates in mTeSR1 medium (Stem Cell Technologies) with daily feeding in 37°C/5% CO_2_/85% humidity.

Pluripotency for all iPSC lines was confirmed by immunocytochemistry using antibodies (Ab) against Tra-1-60, Tra-1-81, SSEA3 and SSEA4, which are expressed in pluripotent stem cells. In addition, the capacity to differentiate into all 3 germ layers was established by *in vitro* assays, as previously described [63,64]. The markers desmin (mesoderm), α-fetoprotein (endoderm), and βIII-tubulin (ectoderm) were used [70–73]. A list of the antibodies used to evaluate the iPSCs can be found in S1 Text. Karyotyping was carried out by Cell Line Genetics (Madison WI). Each iPSC line used in this study had a normal G-banded karyotype, which was used to screen for gross chromosomal changes that can occur during iPSC development. The 3 Mb deletion on 22q11.2 in the patient samples was identified by FISH using a TUPLE probe or microarray. Subjects were matched for age (control mean +/- standard deviation = 37.2+/-12.2; patients; 31.7+/-6.4, *p* = 0.35, Student’s *t*-test, two tailed). Due to technical issues there was only one female subject in the control group and three in the patient group. However, to compensate for this discrepancy, three different clones were analyzed in the control female.

### Neuronal Differentiation

Neurons were generated from iPSC-derived neural progenitor cells (NPCs) as described by Marchetto et al. with slight modifications [63,65]. A detailed description of the protocol can be found S1 Text. Essentially, the protocol leads to a mixed population of glutamatergic and GABAergic neurons, from which RNA was extracted and sent for sequencing.

### miRNA sequencing and data analysis

Briefly, small RNAs were extracted from day 14 neurons using miRNeasy. Libraries were constructed using NEBNext Multiplex Small RNA Library Prep Kit (Set1 for Illumina) and size selection of the Small RNA library (147 bp) was performed using Pippin Prep instrument using 3% Agarose, dye free gel with internal standards (Sage Science # CDF3010) according to the manufacturer’s instructions. MicroRNA sequencing was carried out using the Illumina HiSeq2500 Massively Parallel Sequencing platform as single end 50 bp read length.

For data analysis, 3’ adaptor sequences (TGGAATTCTCGGGTGCCAAGG) were removed from the raw miRNA-seq reads using a java script “AdRec.jar” from seqbuster [74]. Out of the total, ~7% of reads were without adaptors. 92% of processed reads after adaptor trimming were 15-35bp in length. The trimmed reads were then mapped to known pre-miRNA sequences deposited in miRBase (hsa database from miRBASE20) (http://www.mirbase.org/), allowing for one mismatch at most using bowtie ([75]. For any read to be considered as from a known mature miRNA, its 5’ and 3’ ends needed to be within 1–3 bp from the 5’ and 3’ ends of the mature miRNAs annotated in miRBase, respectively. To avoid mis-mapping, all trimmed reads were also mapped to the human genome reference sequences (hg19). Any reads initially assigned to a mature miRNA was re-assigned as non-miRNA in origin if it had a superior match outside the miRNA locus. Reads not mapped to miRNAs were also annotated and categorized based on their overlap with known gene annotations in the GENCODE (V18) (S1 Fig) [76].

To identify differentially expressed (DE) miRNAs, we applied DESeq2 to analyze the read counts of all microRNAs [77]. Specifically, DESeq2 normalized read counts across samples using size factors, estimated as the median of the ratio of a sample’s observed counts to the geometric mean of counts across samples. It then modeled the variance in miRNA read counts across replicates using the negative binomial distribution and then tested whether, for a given miRNA, the change in counts between the control and SZ/SAD samples was significantly larger as compared to the variation within each replicate group. A nominally significant *p*-value of < 0.01 and fold change >1.5-fold were chosen as the cutoffs for identifying differentially expressed miRNAs between SZ and controls, but a multiple comparison correction was also applied to adjust the *p*-values for genome-wide significance [78,79]. The total number of miRNA-seq reads for each sample is shown in S1 Table. The miRNA-seq data have been deposited in Gene Expression Omnibus (GEO; accession number GSE65367).

### miRNA target gene prediction

The target genes for DE miRNAs were predicted using the Ingenuity Pathway Analysis (IPA) MicroRNA Target Filter, which combines experimentally validated targets from TarBase and miRecords, predicted targets from TargetScan, and manually collected miRNA-mRNA interactions from the peer-reviewed literature [80–83]. To reduce false positive targets, we only took into consideration the targets that were experimentally validated or predicted with high confidence. Then, IPA Core Analysis and the software DAVID were used for function analysis of the target genes [84,85]. A right-tailed Fisher’s exact test was run through IPA software and functions with *p*-value < 0.05 were considered significant. A modified Fisher’s exact test was run through DAVID and functional categories with *p*-value < 0.05 were considered significant [84,85].

### Visualization of functional connections of predicted miRNA targets

To better understand how the significantly altered miRNAs could impact neural and brain function and development, we first used the DAVID analysis to detect biological process GO terms enriched (*p*-value < 0.05) in the targets of each differentially expressed miRNA. The neuron/brain-related GO terms were selected and the miRNA targets in these terms were then used to determine pairwise overlap coefficients [86]. A coefficient > 0.5 was used to connect two GO terms, resulting in a network. To illustrate the relationship between miRNAs and the GO term network, an edge was subsequently added between a specific miRNA and a GO term enriched among the predicted target of this miRNA. We further organized the GO terms into functional groups with reference to QuickGO [87] (http://www.ebi.ac.uk/QuickGO/). The final network was generated in Cytoscape 3.2.0 and exported for further editing of colors and labels in Adobe Illustrator [88].

### Validation of mature miRNAs and *DGCR8* by real time quantitative PCR (qPCR)

Expression levels of selected miRNAs were validated using Mercury LNA Universal RT microRNAs (Exiqon, Woburn, MA). PCR was carried out using an ABI-7900 HT Thermocycler in the presence of ROX (300 nM), which was used as a passive reference. Samples were analyzed in triplicate using the 2^-ΔΔCt^ relative expression method normalized with two small RNAs; *SNORD48* and *U6*. The mean expression values from 4–6 patient and control samples each were determined using both controls as normalizers. To measure the relative expression of *DGCR8*, standard qPCR was carried using beta-2 microglobulin as the normalizing control, as we have previously described [64,84]. For the statistical analysis, the control and SZ relative expression levels were pooled and the mean fold difference (controls vs SZ) was determined. A Student’s t-test was used to determine statistical significance.

## Results

### microRNA sequencing

MicroRNA-seq was carried out on day 14 neurons obtained from iPSCs derived from six controls and six patients with SZ or SAD. For one control sample (ctrl_iPSC1), three independent clones were analyzed, and for another control (ctrl_iPSC2), two different clones were analyzed, resulting in a total of 9 control samples. Among the 22q11.2 del samples, two different clones for SZ_iPSC15 were analyzed resulting in a total of 7 samples. After the miRNA-seq reads were analyzed and annotated, we found that the percentages of small RNA reads originating from miRNAs were, overall, similar among samples, but the SZ samples showed slightly reduced percentages (S1 Fig). The Spearman correlation coefficient was high (>0.8) across the patient samples and controls (S2 Fig).

After normalizing the counts, each sample showed high levels of expression of miRNAs associated with neurogenesis (miR-9, miR-124, miR-125a, miR-125b, miR-181a, miR-219, and let-7; for example), validating the neuronal nature of our samples. More modest levels of expression for other miRNAs involved in neurogenesis were detected, including miR-17, miR-184, miR-132, miR-324-5p, miR-326, and the SZ candidate miR-137. However, there were no significant differences in expression between patients and controls for these particular miRNAs (S2 Table).

**Fig 1:**
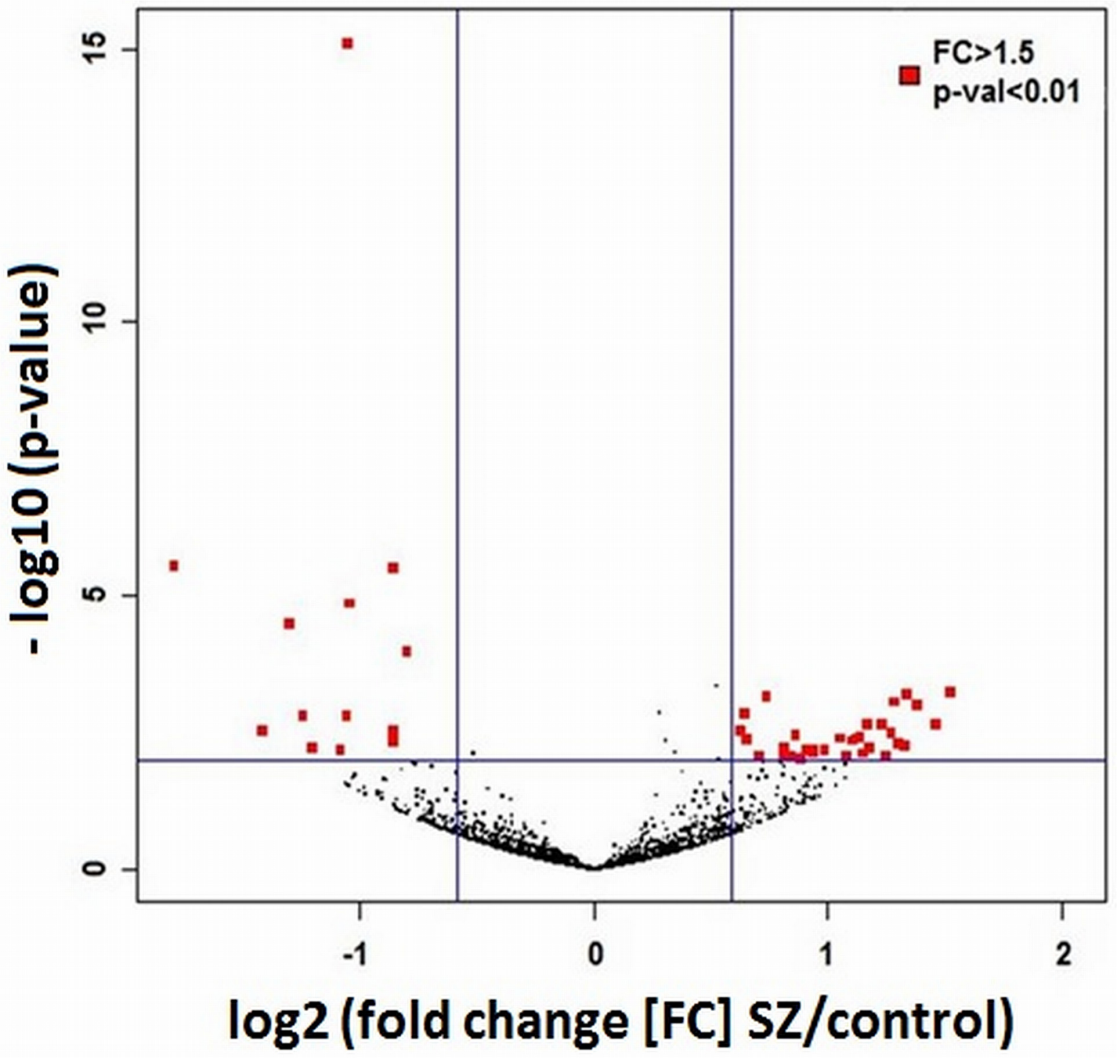
Volcano plot showing statistical significance (-log10 of the *p*-values) on the y-axis vs fold change of all expressed miRNAs. The 13 miRNAs that were significantly down-regulated in SZ are shown at the left (red squares), while the 32 that were significantly up-regulated in SZ are shown at the right (*p* < 0.05 and FC > 1.5).

Using a *p*-value <0.01 and >1.5-fold differences in expression as limits, there were 45 differentially expressed miRNAs (13 lower in SZ; 32 higher; Fig 1, Table 2). Of these, 6 were significantly down-regulated in the 22q11.2 del neurons after correcting for genome wide significance (FDR<0.05), including 4 miRNAs that map to the 22q11.2 del region (miR-1306-3p, miR-1286, miR-1306-5p and miR-185-5p), and two that do not (miR-3175, miR-3158-3p). Two of the down-regulated miRNAs (miR-185 and miR-491) overlapped with the 25 that were found to be similarly down-regulated in the hippocampus and prefrontal cortex in *Dgcr8* knockout mice [31,50].

**Table 2:** Differentially expressed miRNAs.

Down regulated	coordinates	log2 FC	p-value	p-adjusted
miR-1306-3p	chr22:20073635_20073652	1.05	7.52E-16	1.34E-12
miR-1286	chr22:20236668_20236688	1.79	2.89E-06	1.84E-03
miR-1306-5p	chr22:20073595_20073616	0.85	3.09E-06	1.84E-03
miR-185-5p*	chr22:20020676_20020697	1.04	1.37E-05	6.11E-03
miR-3175	chr15:93447638_93447659	1.30	3.12E-05	1.12E-02
miR-3158-3p	chr10:103361184_103361205,chr10:103361223_103361244	0.80	9.97E-05	2.97E-02
miR-185-3p*	chr22:20020711_20020732	1.06	1.49E-03	1.69E-01
miR-486-3p*	chr8:41517961_41517981,chr8:41518004_41518024	1.24	1.51E-03	1.69E-01
miR-1249	chr22:45596839_45596860	0.86	2.96E-03	2.44E-01
miR-6840-5p	chr7:99954279_99954302	1.41	3.00E-03	2.44E-01
miR-491-5p*	chr9:20716119_20716140	0.85	4.63E-03	2.67E-01
miR-4804-5p	chr5:72174427_72174447	1.20	5.88E-03	2.93E-01
miR-767-3p	chrX:151561919_151561941	1.08	6.32E-03	2.93E-01
**Up regulated**	**coordinates**			
miR-34b-3p*	chr11:111383712_111383733	-1.52	5.81E-04	1.21E-01
miR-34c-5p*	chr11:111384176_111384198	-1.33	6.29E-04	1.21E-01
miR-26b-5p*	chr2:219267380_219267400	-0.74	6.75E-04	1.21E-01
miR-146b-3p*	chr10:104196313_104196334	-1.28	8.59E-04	1.40E-01
miR-23a-5p*	chr19:13947444_13947465	-1.38	9.86E-04	1.47E-01
miR-296-3p*	chr20:57392681_57392702	-0.64	1.44E-03	1.69E-01
miR-4449*	chr4:53578887_53578908	-1.46	2.14E-03	2.09E-01
miR-4792	chr3:24562903_24562920	-1.22	2.21E-03	2.09E-01
miR-148a-3p	chr7:25989542_25989563	-1.17	2.22E-03	2.09E-01
miR-320b	chr1:117214409_117214430,chr1:224444751_224444772	-0.63	2.90E-03	2.44E-01
miR-3609	chr7:98479323_98479346	-1.27	3.26E-03	2.54E-01
miR-320c	chr18:19263520_19263539,chr18:21901680_21901699	-0.86	3.52E-03	2.61E-01
miR-126-3p*	chr9:139565105_139565126	-1.13	3.87E-03	2.61E-01
miR-320e	chr19:47212551_47212568	-1.05	3.94E-03	2.61E-01
miR-7704	chr2:177053571_177053589	-1.12	3.94E-03	2.61E-01
miR-181b-5p*	chr1:198828054_198828076,chr9:127456004_127456026	-0.65	4.08E-03	2.61E-01
miR-146a-5p*	chr5:159912379_159912400	-1.10	4.37E-03	2.66E-01
miR-6757-5p	chr12:53450733_53450754	-1.30	5.12E-03	2.86E-01
miR-4682	chr10:121718034_121718056	-1.32	5.59E-03	2.93E-01
miR-26a-5p*	chr12:58218441_58218462,chr3:38010904_38010925	-0.81	6.08E-03	2.93E-01
miR-3195	chr20:60639868_60639884	-1.18	6.16E-03	2.93E-01
miR-126-5p*	chr9:139565068_139565088	-0.94	6.31E-03	2.93E-01
miR-125a-5p	chr19:52196521_52196544	-0.91	6.39E-03	2.93E-01
miR-548q	chr10:12767324_12767345	-0.98	6.66E-03	2.98E-01
miR-320d	chr13:41301964_41301982,chrX:140008337_140008355	-0.94	7.37E-03	3.10E-01
miR-4497	chr12:110271155_110271171	-1.15	7.47E-03	3.10E-01
miR-27a-3p*	chr19:13947261_13947281	-0.84	8.11E-03	3.17E-01
miR-455-5p	chr9:116971729_116971750	-0.70	8.25E-03	3.17E-01
miR-7113-5p	chr11:67800332_67800352	-0.81	8.33E-03	3.17E-01
miR-6842-5p	chr8:27290892_27290913	-1.25	8.64E-03	3.18E-01
miR-146b-5p	chr10:104196277_104196298	-1.07	8.70E-03	3.18E-01
miR-6852-5p	chr9:35710713_35710733	-0.88	9.21E-03	3.29E-01

Asterisk (*) shows miRNAs that have also been found to be differentially expressed in autopsy samples or peripheral cells in neuropsychiatric disorders (see text for references). Abbreviation: FC (fold change)

A total of 7 known miRNA genes map to the large 22q11.2 del region, producing 10 different mature miRNAs, 5 of which are expressed at relatively high levels in our differentiating neurons (miR-1306-3p, miR-1286-3p, and miR-1306-5p, miR-185-5p and miR-185-3p) (S2 Table). Three other mature miRNAs are expressed at relatively low levels (normalized RPM <1; miR-6816-3p, miR-4761-3p and miR-4761-5p), and two are not expressed at all in our neurons: miR-649 and miR-3168, the latter of which is involved in cardiovascular development. Of the 5 mature, highly expressed miRNAs, all showed a significant ~50% decrease in the 22q11.2 del samples compared with controls (miR-1306-3p, miR-1286, miR-1306-5p, miR-185-5p and miR-185-3p; miR-185-5p); four miRNAs were differentially expressed at the genome-wide significance including, miR-185, as noted above (Table 1). By contrast, 41 mature miRNAs that map to chromosome 22 outside of the deleted region are expressed in the neurons (out of total of 67 miRNA genes on chromosome 22). Of these, only one (miR-1249) showed a nominally significant difference compared to control neurons. The difference in differentially expressed mature miRNAs in the 22q11.2 del region that showed nominally significant differences in expression compared with mature miRNAs generated from the remainder of miRNA genes on chromosome 22 is highly significant (Fisher exact test, *p* = 2E-06). This shows that neurons derived from iPSCs that carry the 22q11.2 del recapitulate the miRNA expression patterns expected of 22q11.2 haploinsufficiency.

There were no differentially expressed up-regulated miRNAs that achieved genome wide significance. The most significant were miR-34b-3p and miR-34c-5p, which are members of the miR‑34 family. These miRNAs regulate the mitotic cell cycle, cell migration and apoptosis, and one member, miR-34a, has been found to be differentially expressed in patients with SZ and ASD (see discussion) [89–96]. It should be noted that miR-34a-5p showed a 1.6-fold increase in the SZ neurons in our study (S2 Table). However the results did not reach our threshold for nominal significance (*p* = 0.11).

Other miRNAs that show the largest fold increases in the SZ samples were miR-4449, miR-146b-3p, and miR-23a-5p. These are all of interest in neuropsychiatric disorders as described in greater detail in the Discussion section [97–102]. MicroRNA-146 also affects IL-6 expression and regulates inflammatory responses and innate immunity, factors associated with SZ and ASD risk [98,103–108]. These findings show that in addition to down-regulated miRNAs caused by haploinsufficiency for *DGCR8* and the miRNA genes that map to the 22q11.2 del region, increased expression of some miRNAs may also play a role in the pathogenesis of neuropsychiatric disorders associated with 22q11.2 del.

### Validation

**Fig 2:**
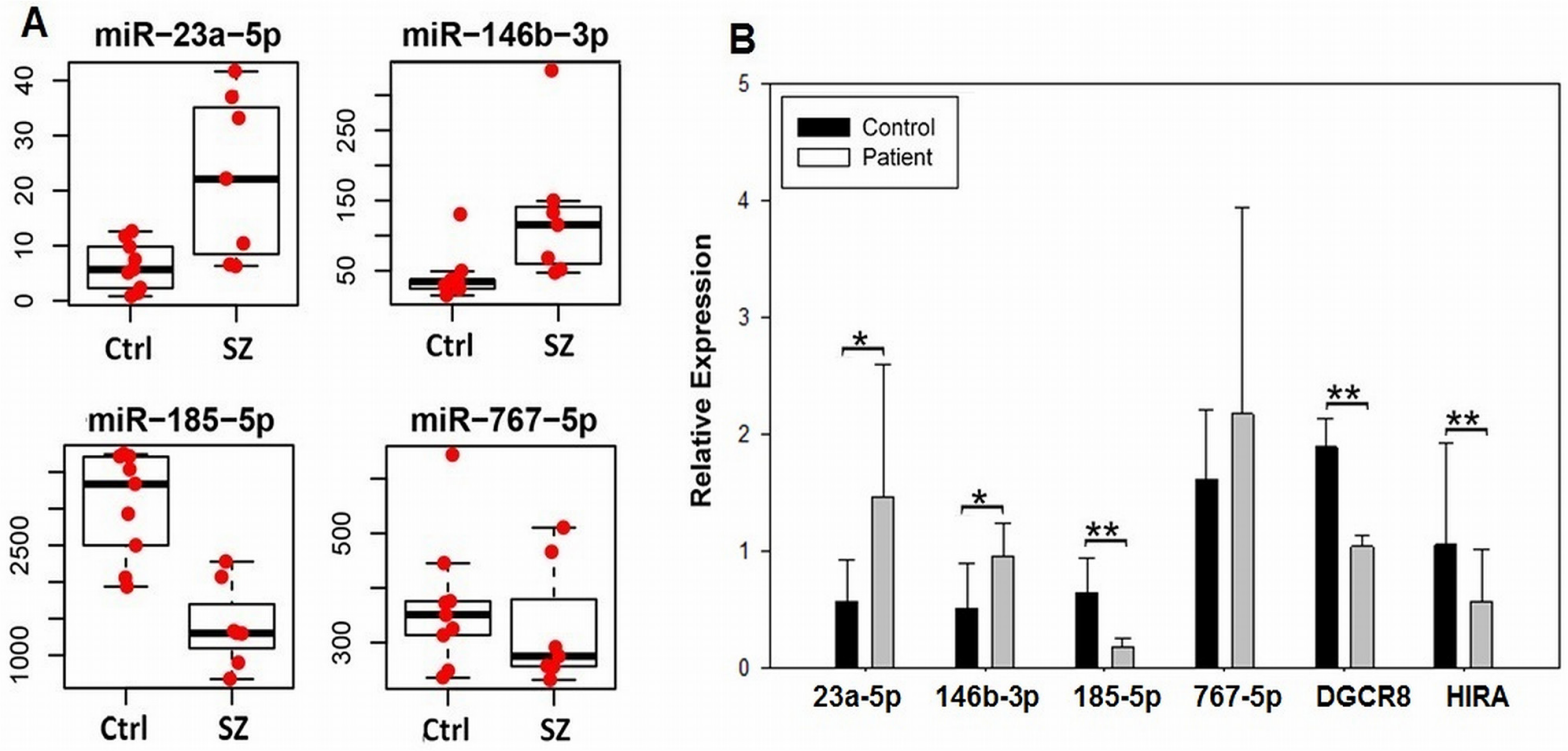
A. miRNA-seq reads (y-axis) for controls (ctrl) and patients with 22q11.2 del (SZ) showing two, nominally significant up-regulated genes (miR-23a-5p and miR-146b-3p), a miRNA that showed a genome wide significant decrease in expression (miR-185-5p), and a miRNA that showed no difference in expression (miR-767-5p). B. Same miRNAs in 2A showing qPCR analysis using Mercury LNA Universal RT microRNAs assays, as described in methods. The relative expression of two coding genes that map to the 22q11.2 del region (*DGCR* and *HIRA*) was analyzed by routine qPCR. A Student’s t-test was used for statistical analysis. Error bars show standard deviation; *p*<0.05, one-tailed*; *p*<0.05, two-tailed.**

The expression of four miRNAs that showed either increased or decreased levels, or no change, in the SZ samples relative to controls was validated by qPCR, as described in the methods section. As shown in Fig 2, fold differences in expression were similar to that seen in the miRNA-seq experiments. In addition, we used qPCR to evaluate the expression of *DGCR8* mRNA in our patient vs control samples. As seen in the figure, the qPCR results support the miRNA-seq findings; a significant decrease in *DGCR8* expression is seen in the patient samples, as expected of a 22q11.2 del disorder. A significant decrease in the expression of *HIRA*, which also maps to the 22q11.2 del region was detected as well.

### Predicted mRNA targets of differentially expressed miRNAs

In order to determine the potential impact of differentially expressed miRNAs on neuronal function, putative gene targets were predicted and functionally characterized as described in the Methods section. A total of 5,413 predicted targets were found with the high confidence setting in IPA among the up-regulated miRNAs, and 2,274 predicted targets were found for the down-regulated miRNAs (S3 Table). These high confidence targets were used in subsequent analyses because the number of experimentally validated targets was too small (240 targets for up-regulated miRNAs; 10 for the down-regulated miRNAs) for pathway analyses. According to IPA, the top two diseases/functions enriched within the predicted targets of both up and down-regulated miRNAs were neurological and psychological disorders (S4 Table; Table 3). Among the predicted targets of up-regulated miRNAs were a number of well-established SZ, ASD and BD candidates, including *DISC1*, *GSK3β*, *MYT1L*, *TCF7L2*, *CNTNAP1*, *NRXN1*, genes involved in glutamatergic transmission (*GRM3*, *GRIN2A*, *GRIN2B*, *GRIN2D*, *GRIK2*, *GRIK3*), and genes involved in GABAergic transmission (*CCK*, *GABRA1 GRIN2B*,*GABBR2*, *GABRB2*). Among the down-regulated miRNA gene targets were the candidate genes *GSK3B*, *CNTNAP1*, *DAO*, *GRIA1*, *GRIN1*, *GRIK3*, *and SLC17A7 (VGLUT1)*.

**Table 3:** Ingenuity Pathway Analysis (IPA): Diseases/Functions of mRNA targets of differentially expressed miRNAs.

**Diseases/Functions: targets of up-regulated miRNAs**	**p-value**
Neurological Disease	6.74E-04-4.49E-02
Psychological Disorders	6.74E-04-4.49E-02
Metabolic Disease	7.39E-04-4.49E-02
Cell Death and Survival	2.18E-03-4.76E-02
Cellular Growth and Proliferation	2.26E-03-3.42E-02
Cellular Development	3.38E-03-4.49E-02
Nervous System Development and Function	3.38E-03-4.49E-02
Tissue Development	3.38E-03-4.49E-02
Tissue Morphology	6.1E-03-4.49E-02
Cellular Assembly and Organization	1.49E-02-3.42E-02
**Disease/Functions: targets of down-regulated miRNAs**	**p-value**
Neurological Disease	1.42E-02-2.95E-02
Psychological Disorders	1.42E-02-2.95E-02
Metabolic Disease	1.66E-02-1.96E-02
Cell Morphology	1.89E-02-1.89E-02
Cellular Function and Maintenance	1.89E-02-1.89E-02
Nervous System Development and Function	1.89E-02-1.89E-02

Ingenuity Pathway Analysis (IPA), Diseases/Functions category of mRNA targets of differentially expressed miRNAs. Table shows top IPA diseases and functions for predicted gene targets.

Similarly, using the DAVID functional annotation tool, SZ was the top disease category (Table 4) for up-regulated miRNA target genes, when the predicted targets were analyzed for enrichment of genes associated with genetic disorders. In addition, other neuropsychiatric disorders previously reported in patients with 22q11.2 del were also among the top diseases, including BD, Tourette Syndrome and SAD, as well as cleft palate, one of the most common physical anomalies associated with VCFS [32–34,36,109,110]. Interestingly, target genes involved in type 2 diabetes were also somewhat enriched. This suggests that there may be some common genetic factors involved in the development of type 2 diabetes, which is seen as part of the “metabolic syndrome” that occurs in a substantial subgroup of SZ patients treated with anti-psychotic medications, consistent with some published reports [111,112].

**Table 4:** DAVID functional annotation; Top Diseases for predicted targets of differentially expressed miRNAs.

**Top Diseases for predicted targets of up-regulated miRNAs**			
Term	Count	%	P-Value
schizophrenia	116	2.29	3.30E-04
Alzheimer's Disease	91	1.79	0.01
colon cancer rectal cancer	6	0.12	0.01
schizophrenia; schizoaffective disorder; bipolar disorder	10	0.20	0.02
prostate cancer	66	1.30	0.02
bipolar affective disorder	8	0.16	0.02
sleep disorders	8	0.16	0.02
cleft lip with cleft palate cleft lip without cleft palate cleft palate	16	0.32	0.03
colorectal cancer; Tourette syndrome; bone density; pregnancy loss, recurrent; cleft lip without cleft palate; juvenile polyposis; cleft palate	6	0.12	0.03
diabetes, type 2 triglycerides	6	0.12	0.03
**Top Diseases for predicted targets of down-regulated miRNAs**			
Term	Count	%	P-Value
myelopathy, HTLV-1 associated	5	0.24	0.003
narcolepsy	7	0.33	0.004
diabetes, type 1	38	1.80	0.004
pulmonary hypertension	4	0.19	0.02
sclerosis, systemic	10	0.47	0.03
HTLV-1 infection	5	0.24	0.04
malaria; schistosomiasis	3	0.14	0.04
migraine; migraine with aura	4	0.19	0.05
normal variation	7	0.33	0.05
diabetes mellitus	5	0.24	0.05
Graves' disease	9	0.43	0.06
bipolar disorder	20	0.95	0.06
schizophrenia	45	2.13	0.07

DAVID functional annotation; Top Diseases for predicted targets of differentially expressed miRNAs.

Genes involved in SZ and BD were also found among the predicted targets of down-regulated miRNAs, although the *p*-values were modest (*p* = 0.06 and 0.07, respectively, Table 4). A number of infectious and autoimmune disorders were among the top diseases in this category as well, consistent with the immune problems found in patients with 22q11.2 del. Whether this also influences the underlying autoimmune and/or infectious disease etiology believed to play a role in the pathogenesis of SZ and ASD in a subgroup of patients remains to be determined [54,55,113–116].

The predicted target genes were also characterized by Gene Ontology (GO). As seen in Table 5, the most enriched GO terms in the cellular component (CC) assessment were synapse and neuron projection for predicted targets of up-regulated miRNAs; these were also among the top hits for predicted targets of down-regulated miRNAs (see S5 Table and S6 Table for details). Abnormal synaptogenesis and neuron projection are primary pathogenic features of both SZ and ASD [117–125].

**Table 5:** DAVID functional annotation; Gene Ontology (GO) categories (CC).

**GO categories**	**Count**	**%**	**p-value**	**FE**	**Bonferroni**	**Benjamini**	**FDR**
GO:0045202~synapse	172	3.39	1.23E-18	1.81	9.56E-16	9.56E-16	1.88E-15
GO:0043005~neuron projection	167	3.29	1.54E-18	1.82	1.20E-15	6.02E-16	2.36E-15
GO:0005794~Golgi apparatus	332	6.55	4.56E-14	1.42	3.56E-11	1.19E-11	6.98E-11
GO:0042995~cell projection	269	5.30	2.87E-12	1.44	2.24E-09	5.60E-10	4.39E-09
GO:0005626~insoluble fraction	313	6.17	4.99E-12	1.39	3.89E-09	7.79E-10	7.64E-09
GO:0005624~membrane fraction	302	5.95	1.15E-11	1.39	8.95E-09	1.49E-09	1.76E-08
GO:0030424~axon	82	1.62	6.01E-11	1.93	4.69E-08	6.70E-09	9.20E-08
GO:0000267~cell fraction	383	7.55	6.93E-11	1.32	5.40E-08	6.75E-09	1.06E-07
GO:0019898~extrinsic to membrane	197	3.88	1.31E-10	1.49	1.02E-07	1.13E-08	2.00E-07
GO:0044459~plasma membrane part	711	14.02	1.95E-10	1.21	1.52E-07	1.52E-08	2.98E-07
**GO categories**	**Count**	**%**	**p-value**	**FE**	**Bonferroni**	**Benjamini**	**FDR**
GO:0044459~plasma membrane part	356	16.84	9.98E-13	1.40	5.97E-10	5.97E-10	1.47E-09
GO:0005886~plasma membrane	519	24.55	7.08E-07	1.19	4.23E-04	2.12E-04	1.05E-03
GO:0045202~synapse	73	3.45	1.11E-06	1.78	6.61E-04	2.21E-04	1.63E-03
GO:0042995~cell projection	121	5.72	3.54E-06	1.50	0.00	5.29E-04	0.01
GO:0043005~neuron projection	69	3.26	4.52E-06	1.74	0.00	5.40E-04	0.01
GO:0045121~membrane raft	36	1.70	1.12E-05	2.18	0.01	0.00	0.02
GO:0031226~intrinsic to plasma membrane	184	8.70	6.27E-05	1.31	0.04	0.01	0.09
GO:0005887~integral to plasma membrane	180	8.51	7.38E-05	1.31	0.04	0.01	0.11
GO:0005624~membrane fraction	129	6.10	1.06E-04	1.38	0.06	0.01	0.16

DAVID functional annotation for predicted targets of differentially expressed miRNAs (see S5 Table and S6 Table for details). GO categories CC (cellular components) based on predicted targets of up and down-regulated miRNAs

### Network analysis

**Fig 3:**
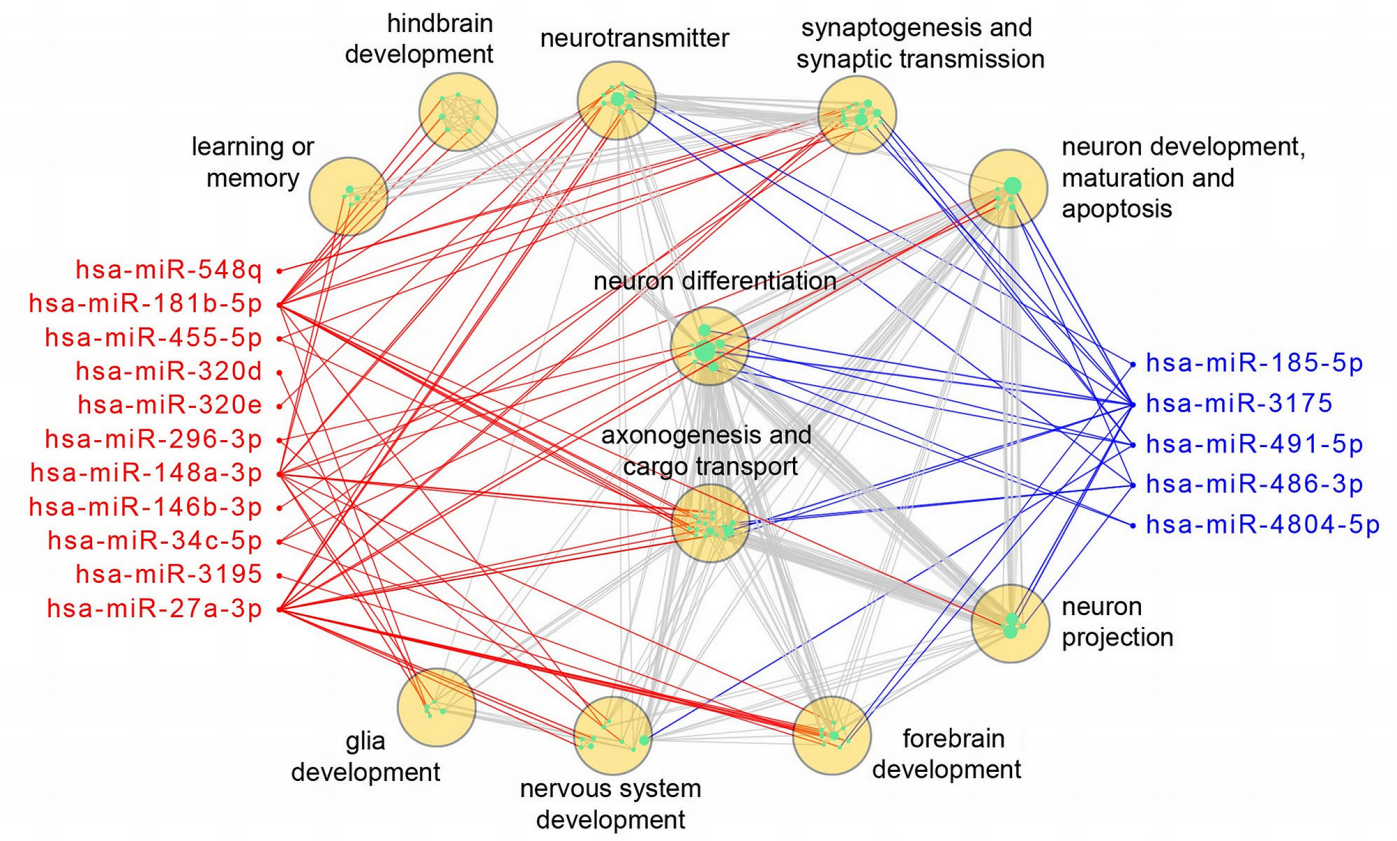
A network view of neurological function of significantly altered miRNAs. Red nodes stand for up-regulated miRNAs, blue for down-regulated miRNAs, and cyan for enriched GO terms (n = 91, with sizes proportional to the numbers of predicted miRNA targets). GO terms were further classified into broader function groups (yellow circles) by QuickGO. A red or blue edge indicates a GO term was enriched in the predicted targets of up-regulated or down-regulated miRNAs, respectively. Grey edges link GO terms with overlapped targets (overlap coefficient > 0.5). This network was created by the Cytoscape 3.2.0.

To better illustrate how the differentially expressed miRNAs may independently or cooperatively affect brain development and function, we constructed a network of differentially expressed miRNAs and the enriched neural GO terms of their putative targets, as described in the Methods section [86,87] (Fig 3). The data show that 11 up-regulated and 5 down-regulated miRNAs can regulate a broad range of brain related genes, with miR-181b-5p, miR148a-3p, miR-27a-3p, and miR-3175 potentially having the largest impact. The result also indicates that genes involved in neurotransmitter function, synaptogenesis, and neuronal differentiation will likely be affected most by the disruption of the miRNAs found in our 22q11.2 del neurons. Also, while forebrain development can be affected by both up- and down-regulated miRNAs in SZ, hindbrain development seems be affected more by the up-regulated miRNAs. The figure reinforces our finding that in differentiating neurons, up-regulated, as well as down-regulated miRNAs and their targets are affected by 22q11.2 del, and consequently can affect multiple processes in brain development and function.

## Discussion

Research on the potential role of miRNAs in 22q11.2 del-associated neuropsychiatric disorders has primarily focused on analyzing the effects of *DGCR8* on miRNA expression and behavior, which stands to reason considering the effect it has on miRNA biogenesis [31,48,117]. More recently, there have been studies showing the effects of specific miRNAs that map to the deleted region, most notably *MIR-185*, which may influence neuronal function and synaptogenesis independently of DGCR8-mediated biogenesis [7,49,51,117]. Overall, the bioinformatics analysis of predicted targets of miRNAs that were found to be down-regulated in our 22q11.2 del samples, which included miR-185, is consistent with the mouse studies in that there was enrichment for genes involved in neuropsychiatric disorders, synaptogenesis and neuron projection. However, without direct functional studies, the relative contribution of DGCR8, miR-185 and other down-regulated miRNAs, some of which are affected by reduced levels of DGCR8, remains uncertain.

There were two miRNAs that were significantly down-regulated in our 22q11.2 neurons that overlapped with the 25 that were found in *Dgcr8* knockouts; miR-185 and miR-491[31]. The reason for the small degree of overlap is not clear, but could be due to species differences, cellular heterogeneity in the neuroanatomical structures analyzed in mice, which could potentially amplify an effect in non-neuronal cells, and the relative immaturity and spatio-temporal differences of the neurons used in our analysis. Although miR-185 has established effects on neuronal function, as noted above, less is known about miR-491 in the context of neuronal function and disease pathogenesis. However, in cancer cells, expression is correlated with an increase in apoptosis through activation of intrinsic mitochondrial apoptotic pathways, and cell growth is affected by the inhibitory effect of miR-491 on PI3K/Akt signaling [126]. Abnormalities in apoptosis and PI3K/Akt signaling have been described in SZ and ASD [127–131].

As for other down-regulated miRNAs that map to 22q11.2, there are no reported functional studies, so their potential role in neuropsychiatric disorders is uncertain. The same can be said for the down-regulated miRNAs detected in our study that map to other chromosomes, with the exception of miR-486 and miR-491. miR-486 was the top hit in an analysis of differentially expressed genes carried out in discordant siblings for ASD using lymphoblastoid cell lines (although expression was higher in the ASD subjects, and lower in our 22q11.2 del neurons) [132]. MicroRNA-486 is known to regulate Akt signaling by targeting *PTEN*, the latter of which is a well-established ASD candidate gene [133–135]. MicroRNA-491 is one of several miRNAs that may affect impulsivity and co-morbid traits associated with synaptic plasticity in the mouse amygdala [136]. This could be of translational interest because amygdala size has been reported to be abnormal in 22q11.2 del [137,138]. Functional **a**bnormalities in the amygdala probably plays some role in the high rate of anxiety disorder seen in patients with 22q11.2 del, as well as their tendency towards impulsivity [39,40,139]. Down-regulation of these miRNAs is likely not due to *DGCR8* haploinsufficiency, at least not entirely, since they were not identified as such in *Dgcr8* knockout mice [31].

Thus, in 22q11.2 del, some down-regulated miRNAs that may play a role in disease pathogenesis are regulated independently of *DGCR8*, their expression affected perhaps by one or more of the transcriptional and chromatin regulators that map to this region of the genome.

An effect on miRNA expression in 22q11.2 del that is independent of DGCR8 is supported by the finding that 32 differentially expressed miRNAs were up-regulated in the 22q11.2 del samples, rather than down-regulated, a number of which have previously been connected to neuropsychiatric disorders. The miR-34 family of related miRNAs is an example. A key member of the miR-34 family is miR-34a, which plays a role in neural stem cell differentiation [93]. Most interestingly, miR-34a is expressed at higher levels in the peripheral blood mononuclear cells and in the prefrontal cortex in SZ patients compared to controls [92,94,95]. Similarly, miR-34a, 34b, and 34c are upregulated in the hippocampus of *Fmr1* KO mice: a CGG trinucleotide repeat in the *FMR1* gene causes Fragile X syndrome [140]. A number of ASD, SZ and BD candidate genes are predicted targets of miR-34c, including *CNTNAP1*, *CNTNAP2*, *GABRA3*, *RELN*, *FOXP2*, *NRXN2*, *and ANK3* (S3 Table).

Finally miR-34a was identified as a hub molecule in a bioinformatics analysis of CNVs in ASD [96].

Another up-regulated miRNA of interest is miR-4449; a significant increase in expression was detected in Brodmann area 46 in SZ autopsy samples, and a trend towards a significant increase was found in the hair follicles of living patients [97]. Others are three members of the miR-146 family and miR-296. MicroRNA-146a and miR-146b were two of the top differentially expressed miRNAs in an animal model of Rett syndrome, although it is lower in the *Mecp2* knockouts, while miR-296 is expressed at significantly higher levels, similar to our SZ neuronal samples [141]. MicroRNA-146 expression is modulated by neuronal activity, and affects IL-6 expression, inflammatory responses and innate immunity, factors associated with SZ and ASD risk [98,103–108]. This is consistent with the finding that predicted targets of both miR-146a-5p and miR-146b-3p are multiple members of the interleukin signaling cascade (S3 Table).

Another suggestive up-regulated miRNA is miR-23a, the expression of which is increased in the cerebellum and transformed lymphoblasts of patients with ASD ([99,100]. Expression is also increased (as is miR-146a) in the hippocampus of patients with epilepsy [102]. Epilepsy is found in a subgroup of patients with 22q11.2 del and shares common genetic risk factors with SZ and ASD at multiple loci [142–147]. Finally, the expression of miR-26a, miR-27a-3p, miR-181b and miR-26b is higher in the DLPFC in SZ [101]. A number of SZ, ASD and bipolar disorder candidates are also high confidence predicted targets of these genes, including *JARID2*, *RGS4*, *TCF7L2*, *GSK3B*, *GABRB3*, *FOXP2*, *CACNA1C*, *NRXN1*, *ANK3*, *CNTNAP2*, among others (S3 Table).

Identifying differentially expressed miRNAs in our *in vitro* model for 22q11.2 del-associated neuropsychiatric disorders is extremely important from the standpoint of interpreting miRNA expression data uncovered using autopsy specimens. Although informative findings have emerged from the analysis of mRNAs and miRNAs using this resource, it is an imperfect system from a variety of perspectives. In addition to the technical challenges associated with using autopsy samples (e.g., brain pH, premorbid medical issues, cause of death, postmortem delay, cellular heterogeneity), there are pathophysiological considerations as well. Patients with SZ, for example, are exposed to a number of environmental factors that can each influence gene expression, such as medications, alcohol and nicotine use, and illicit drugs. The differential expression of mRNAs and miRNAs found in expression profiling studies could easily be influenced by one or more of these factors. A similar set of circumstances could also influence the interpretation of gene expression studies in ASD autopsy samples. In addition, the neurodevelopmental underpinnings of both SZ and ASD predate gene expression changes found in autopsy samples by decades. The finding that a number of differentially expressed miRNAs we detected in iPSC-derived neurons grown under controlled conditions overlap with those found in autopsy samples, supports their involvement in neuropsychiatric disorders. This provides a strong rationale for their more extensive analysis in animal and *in vitro* models.

The same principle applies to the use of peripheral, non-neuronal cells, which are exposed to the same confounding environmental factors, and suffer from the additional shortcoming of being a very imperfect proxy for explaining pathophysiological processes involved in neurodevelopmental disorders. However, peripheral cells are excellent sources of potential molecular biomarkers, as long as a connection can be established between those biomarkers and neuronal function and/or behavior. The finding of common differentially expressed miRNAs in peripheral cells and our cultured neurons, such as the miR-34 family, miR-4449 and miR-23a, supports the idea that they should be evaluated as potential biomarkers to assess clinically relevant phenotypes.

Finally, the finding of common differentially expressed miRNAs in neurons containing a 22q11.2 del and clinical/autopsy samples drawn from the general population of SZ and ASD patients suggests that there are underlying molecular genetic networks shared by 22q11.2 del and candidate genes at other loci.

One caveat to our findings that needs to be discussed is that our analysis was carried out using a mix of phenotypes that included both COS and patients who developed psychotic symptoms during adolescence and adulthood. In addition, we used independent replicate clones for two of the controls and one 22q11.2 del subject. Consequently, we repeated the differential expression analysis without the COS subjects, and again, without the independent clones. Using our threshold for nominal significance (p-value of < 0.01 and fold change >1.5-fold), 26 out of 45 differentially expressed miRNAs fell below the level of significance when the data were analyzed without the COS samples (S7 Table). Similarly, without the replicate samples, 13 differentially miRNAs fell below the threshold. However, the dropouts are due to a small increase in p-values. In the COS samples, 23/26 remained below 0.05 (that is, between 0.01–0.05), and the highest was 0.08. In addition, the fold changes comparing patients vs controls are largely unaffected. Indeed, 17 of the miRNAs that dropped below the threshold showed an increase in the fold change—although p-values increased because of higher sample to sample variability. For the 13 differentially expressed miRNAs that fell below the significance threshold when replicates were removed, all but 2 had p-values between 0.01 and 0.05. In addition, the fold change increased in 10 samples, although, as in the COS samples, the p-values increased because of sample to sample variability. This reanalysis indicates that omitting the COS and replicate samples does not significantly change the major conclusions drawn from our study, and that we need to include all samples to maximize statistical power.

To summarize, we show that iPSCs derived from patients with SZ who have 22q11.2 del is a good model system to study the neuropsychiatric manifestations of this condition *in vitro*. Furthermore, expression profiling shows changes in the expression of several miRNAs that are similar to those found in clinical samples, supporting a role in SZ and ASD pathogenesis and their utility as peripheral biomarkers.

## Supporting Information

S1 Fig The statistics of miRNA-seq reads mapped to different types of annotated RNAs. (TIF)

S2 Fig Spearman correlation coefficient for miRNA-seq for each SZ and control sample. (TIF)

S1 Table Number of miRNA-seq reads in each sample. (DOCX)

S2 Table Normalized miRNA-seq reads for all human miRNAs arranged by order of statistical significance. (XLSX)

S3 Table List of high confidence predicted mRNA targets of miRNAs up-regulated and down-regulated in 22q11.2 SZ neurons. (XLSX)

S4 Table IPA disease/function analysis of the predicted miRNA targets. (XLS)

S5 Table DAVID GO analysis of targets of up-regulated miRNAs. (XLSX)

S6 Table DAVID GO analysis of targets of down-regulated miRNAs. (XLSX)

S7 Table miRNAs from Table 2 that fell below threshold of significance after removing the COS samples or duplicate samples. (XLSX)

S1 Text Detailed methods and clinical descriptions. (DOCX)
