# Supplementary figures and images for: MicroRNA Profiling of Neurons Generated Using Induced Pluripotent Stem Cells Derived from Patients with Schizophrenia and Schizoaffective Disorder, and 22q11.2 Del
Source: PLoS One. 2015 Jul 14;10(7):e0132387. doi: 10.1371/journal.pone.0132387 (PMC4501820; doi:10.1371/journal.pone.0132387)

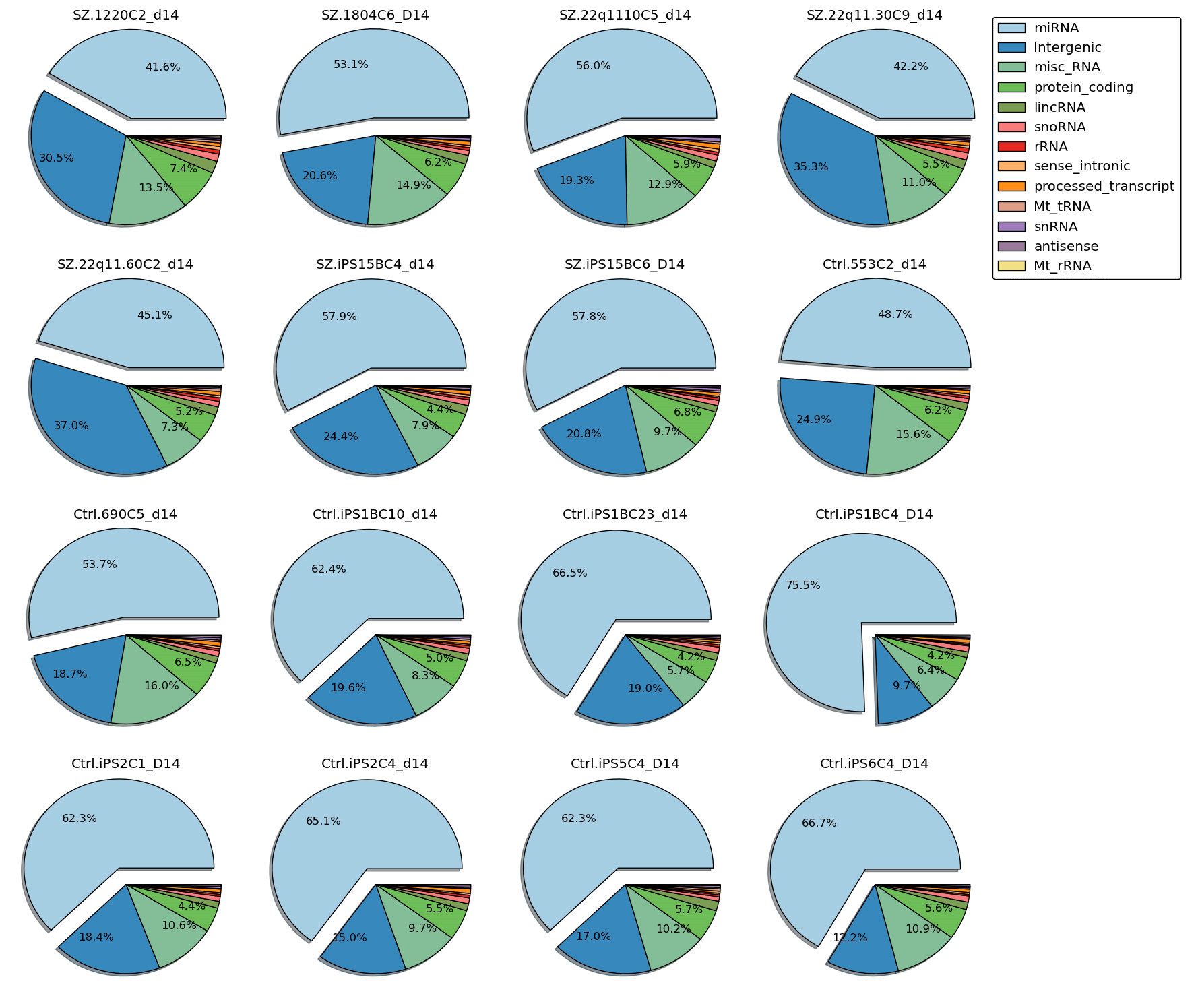

Supplement: S1 Fig — (TIF) [file pone.0132387.s001.tif]

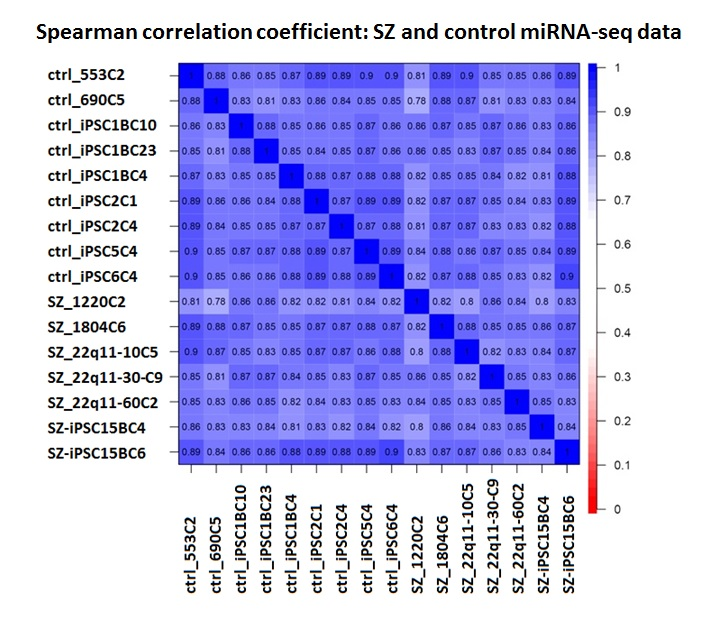

Supplement: S2 Fig — (TIF) [file pone.0132387.s002.tif]
