# Supplementary material for: MicroRNA Profiling of Neurons Generated Using Induced Pluripotent Stem Cells Derived from Patients with Schizophrenia and Schizoaffective Disorder, and 22q11.2 Del
Source: PLoS One. 2015 Jul 14;10(7):e0132387. doi: 10.1371/journal.pone.0132387 (PMC4501820; doi:10.1371/journal.pone.0132387)
Supplement: S1 Table — (DOCX) [file pone.0132387.s003.docx]

| **Subject ID** | **# of raw reads** | **# of reads mapped to genome** | **# of reads mapped to Pre-miRNA** | **# of reads mapped to Mature-miRNA** |
| --- | --- | --- | --- | --- |
| SZ_1220C2 | 8774142 | 6279005 | 2611006 | 2584061 |
| SZ_1804C6 | 9740650 | 7627021 | 4046637 | 4013262 |
| SZ_22q11-10C5 | 9530238 | 7851754 | 4398660 | 4367393 |
| SZ_22q11-30C9 | 16053674 | 12149650 | 5121453 | 5077899 |
| SZ_22q11-60C2 | 13811629 | 11318200 | 5106624 | 5061273 |
| SZ_iPSC15BC4 | 11223856 | 9503664 | 5505642 | 5476304 |
| SZ_iPCS15BC6 | 14955943 | 10622055 | 6138512 | 6084528 |
| Ctrl_553C2 | 20584002 | 16935800 | 8246526 | 8185828 |
| Ctrl_690C5 | 6508122 | 5367999 | 2884858 | 2859700 |
| Ctrl_iPSC1BC10 | 6965019 | 5766106 | 3600026 | 3573503 |
| Ctrl_iPSC1BC23 | 9393294 | 8100108 | 5382383 | 5344771 |
| Ctrl_iPSC1BC4 | 7222641 | 5763227 | 4353669 | 4325196 |
| Ctrl_iPSC2C1 | 9910355 | 8051164 | 5017264 | 4987423 |
| Ctrl_iPSC2C4 | 7970417 | 5725620 | 3726257 | 3696155 |
| Ctrl_iPSC5C4 | 18381758 | 15319093 | 9546596 | 9475235 |
| Ctrl_iPSC6C4 | 20134645 | 17138728 | 11438917 | 11358166 |

**Supporting Information Table S1. Number of miRNA-seq reads in each sample**
